# EBF1 is a potential biomarker for predicting progression from mild cognitive impairment to Alzheimer's disease: an *in silico* study

**DOI:** 10.3389/fnagi.2024.1397696

**Published:** 2024-09-13

**Authors:** Yanxiu Ju, Songtao Li, Xiangyi Kong, Qing Zhao

**Affiliations:** ^1^Department of Neurology, China-Japan Union Hospital of Jilin University, Changchun, China; ^2^Engineering Laboratory of Memory and Cognitive Impairment Disease of Jilin Province, China-Japan Union Hospital of Jilin University, Changchun, China; ^3^Key Laboratory of Lymphatic Surgery of Jilin Province, China-Japan Union Hospital of Jilin University, Changchun, China

**Keywords:** mild cognitive impairment, Alzheimer's disease, nomogram, EBF1, B cells

## Abstract

**Introduction:**

The prediction of progression from mild cognitive impairment (MCI) to Alzheimer's disease (AD) is an important clinical challenge. This study aimed to identify the independent risk factors and develop a nomogram model that can predict progression from MCI to AD.

**Methods:**

Data of 141 patients with MCI were obtained from the Alzheimer's Disease Neuroimaging Initiative (ADNI) database. We set a follow-up time of 72 months and defined patients as stable MCI (sMCI) or progressive MCI (pMCI) according to whether or not the progression of MCI to AD occurred. We identified and screened independent risk factors by utilizing weighted gene co-expression network analysis (WGCNA), where we obtained 14,893 genes after data preprocessing and selected the soft threshold β = 7 at an *R*^2^ of 0.85 to achieve a scale-free network. A total of 14 modules were discovered, with the midnightblue module having a strong association with the prognosis of MCI. Using machine learning strategies, which included the least absolute selection and shrinkage operator and support vector machine-recursive feature elimination; and the Cox proportional-hazards model, which included univariate and multivariable analyses, we identified and screened independent risk factors. Subsequently, we developed a nomogram model for predicting the progression from MCI to AD. The performance of our nomogram was evaluated by the C-index, calibration curve, and decision curve analysis (DCA). Bioinformatics analysis and immune infiltration analysis were conducted to clarify the function of early B cell factor 1 (EBF1).

**Results:**

First, the results showed that 40 differentially expressed genes (DEGs) related to the prognosis of MCI were generated by weighted gene co-expression network analysis. Second, five hub variables were obtained through the abovementioned machine learning strategies. Third, a low Montreal Cognitive Assessment (MoCA) score [hazard ratio (HR): 4.258, 95% confidence interval (CI): 1.994–9.091] and low EBF1 expression (hazard ratio: 3.454, 95% confidence interval: 1.813–6.579) were identified as the independent risk factors through the Cox proportional-hazards regression analysis. Finally, we developed a nomogram model including the MoCA score, EBF1, and potential confounders (age and gender). By evaluating our nomogram model and validating it in both internal and external validation sets, we demonstrated that our nomogram model exhibits excellent predictive performance. Through the Gene Ontology (GO) enrichment analysis, Kyoto Encyclopedia of Genes Genomes (KEGG) functional enrichment analysis, and immune infiltration analysis, we found that the role of EBF1 in MCI was closely related to B cells.

**Conclusion:**

EBF1, as a B cell-specific transcription factor, may be a key target for predicting progression from MCI to AD. Our nomogram model was able to provide personalized risk factors for the progression from MCI to AD after evaluation and validation.

## 1 Introduction

Alzheimer's disease (AD) is a severe neurodegenerative disease, with symptoms of progressive cognitive dysfunction and behavioral impairment. It can lead to diminished quality of life or disability in patients. Due to the unclear cause of the disease and the absence of therapy, early identification and appropriate preventive measures are important. Mild cognitive impairment (MCI) is an intermediate state between normal aging and dementia (Vega and Newhouse, 2014). Due to the high risk of MCI progressing to AD, patients with MCI will be a target for future disease treatments. Therefore, it is necessary to have knowledge about biomarkers and risk factors that can predict the progression from MCI to AD. With the development of biomarkers, it is possible to detect the core pathological changes, even in the preclinical stage of AD. Therefore, there has been a shift in the focus of diagnosis from clinical symptoms to the biomarker framework (Jack et al., 2018). However, the whole spectrum of AD pathologies is not covered by the amyloid-tau-neurodegeneration (A-T-N) framework. Cerebrospinal fluid tests and positron emission tomography (PET) examinations based on this framework have become widely accepted; however, the application of these diagnostic methods is limited due to invasiveness and high cost. Therefore, researchers, including Guo, have proposed adding an “X” to the A-T-N framework, which represents biomarkers of neuroinflammation, neuroimmunity, systemic immunity, and other pathologies, and have focused on peripheral biomarkers (Huang et al., 2022).

A growing number of studies have demonstrated the involvement of the immune system in the pathogenesis of AD (Marsh et al., 2016; Song et al., 2022). Bulati et al. (2015) observed a reduction in the number of B cells in the blood of AD patients, which strongly correlated with patients' Clinical Dementia Rating scores. In APP/PS1 transgenic mice, early B cell depletion significantly accelerated cognitive dysfunction and Aβ burden (Xiong et al., 2021). Similarly, Feng et al. (2023) observed that B cell depletion exacerbated spatial learning and memory deficits in 5 × FAD mice, which was associated with increased Aβ load, reactive gliosis, and synapse-associated protein loss. These data emphasize the neuroprotective role of B cells in AD. Early B cell factor 1 (EBF1) is a B cell-specific transcription factor. It is also involved in the differentiation of the cranial neural crest cells (El-Magd et al., 2014a) and the promotion of neuronal differentiation (Faedo et al., 2017). In our study, we found that EBF1 may be a potential biomarker for predicting the progression from MCI to AD, which provides powerful data for the involvement of B cells in the development of AD.

An accurate prediction of the progression from MCI to AD is crucial for early clinical identification of people at high risk of AD and for effective interventions and treatment to delay its onset. Obtaining reliable biomarkers through simple, effective, low-cost, and non-invasive screening methods has become a hot research topic. In addition to β-amyloid deposition, pathologic tau, and neurodegeneration, we should also focus on the “X.” Currently, studies on EBF1 involve central nervous system disorders, such as multiple sclerosis (Martínez et al., 2005) and Parkinson's disease (Yin et al., 2009). Although it has been shown that EBF1 expression is decreased in the brain with AD, which affects the transcriptional level of FAM3C and promotes Aβ deposition (Watanabe et al., 2021), there are very few studies on the association of EBF1 with AD. In addition, with the popularity of transcriptomics, the amount of biological data has increased exponentially. Bioinformatics methods have been developed rapidly to fully exploit the potential value of these high-throughput data, while machine learning strategies have been widely used in identifying important genes. Based on the above research background, we comprehensively analyzed RNA microarrays through bioinformatics methods, combined with machine learning strategies, to identify the feature gene for progression from MCI to AD. In addition, we developed a nomogram prognostic model to assist physicians in predicting the regression of patients with MCI and to help patients visualize their likelihood of disease development.

## 2 Materials and methods

### 2.1 Participants

The data utilized for our study were acquired from the Alzheimer's Disease Neuroimaging Initiative (ADNI) database (https://adni.loni.usc.edu/). The ADNI was established in 2003 as a collaboration between public and private entities, with approval from the institutional review boards at all ADNI sites. The complete list can be found at http://adni.loni.usc.edu. The primary aim of the ADNI was to test whether it is possible to combine different indicators for predicting the progression of MCI to AD. All participants provided explicit consent before taking part in the study. All procedures were carried out in accordance with the applicable rules and regulations. The Publication Committee of the ADNI approved this study.

According to the ADNI protocol, in the study, MCI was identified when a patient or caregiver reported cognitive decline, if the participant showed indications of impairment in the logical memory II subtest of the Wechsler Memory Scale, if the participant achieved a Mini-Mental State Examination (MMSE) score of 24 or higher, and if the participant had a clinical dementia rating of 0.5. Participants with MCI who met the dementia diagnostic criteria were excluded. AD was diagnosed using the National Institute of Neurological and Communicative Disorders and Stroke (NINCDS) and the Alzheimer's Disease and Related Disorders Association (ADRDA) criteria for probable AD (see http://adni.loni.usc.edu/methods/documents for detailed inclusion and exclusion criteria).

We downloaded 744 participants' peripheral blood RNA microarrays (normalized by the robust multi-chip average method) from the ADNI database. The source of the microarray was GPL13667 (Affymetrix GPL platform), Human Genome U219 Array (see https://ida.loni.usc.edu/pages/access/studyData.jsp for details on RNA microarrays and data preprocessing). Among the744 participants, 383 were patients with MCI.

We set a follow-up time of 72 months, with AD conversion as the endpoint event. We defined the occurrence of the endpoint events as the follow-up endpoint and had the follow-up deadline as the endpoint for those who did not experience the endpoint event. A total of 82 patients with MCI had the endpoint events during the follow-up period and were included in the progressive MCI (pMCI) group, and 59 patients with MCI had no endpoint events within 6 years and were included in the stable MCI (sMCI) group. These patients were included in two cohort studies, ADNI 2 and ADNI GO, with the follow-up period ranging from 6 to 72 months.

**Figure 1:**
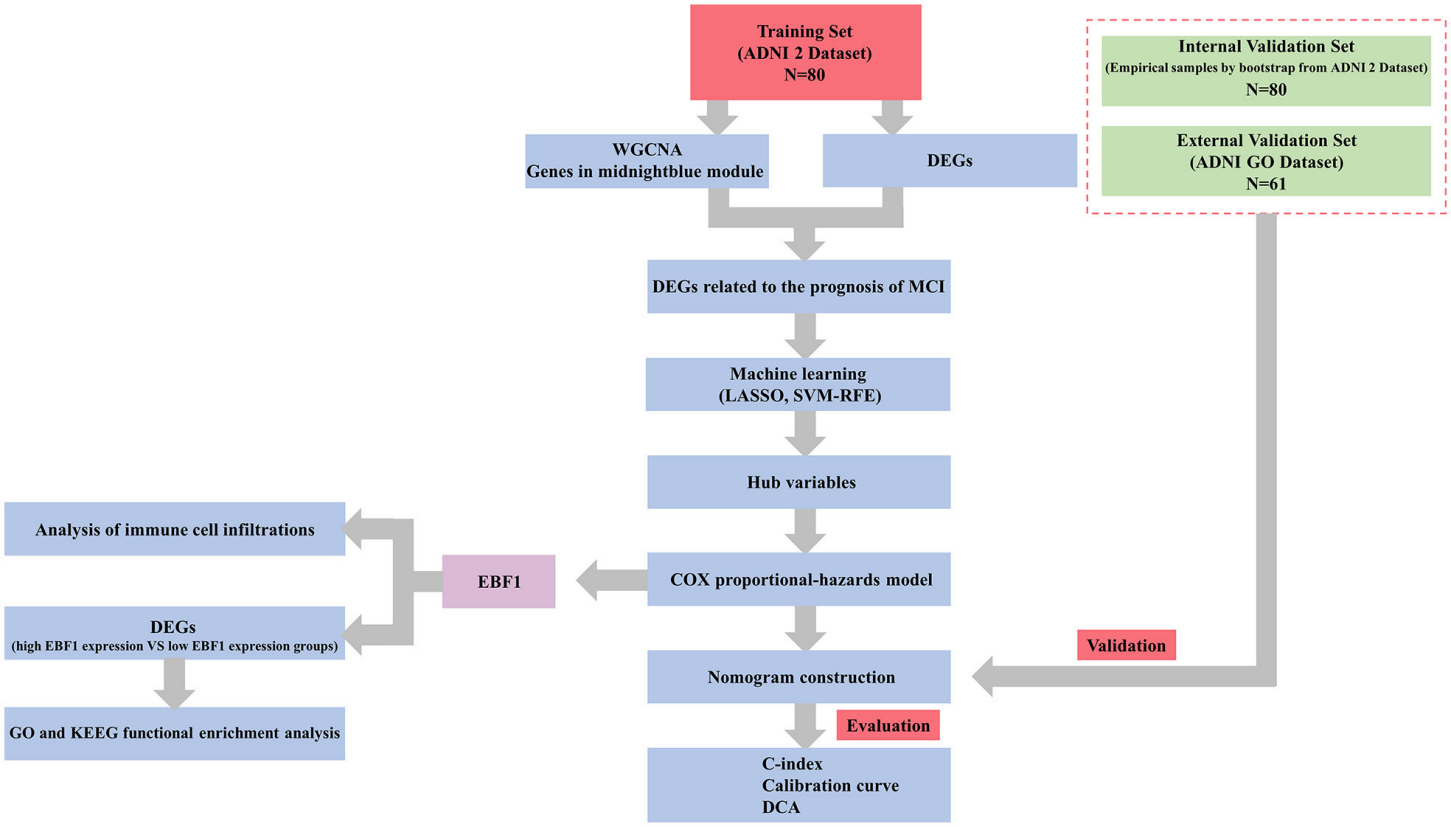
Study flowchart.

In our study, the ADNI 2 dataset (51 cases of pMCI and 29 cases of sMCI) was used for the identification of feature genes, the development of a nomogram prognostic model, and internal validation, whereas the ADNI GO dataset (31 cases of pMCI and 30 cases of sMCI) was used for external validation. According to the study protocol, the remaining patients with MCI were excluded for the following reasons: 11 patients with MCI had outlier RNA microarray samples at the time of weighted gene co-expression network analysis (WGCNA), 29 patients with MCI were converted to cognitively normal cohort (CN) at the follow-up endpoint, 169 patients with MCI were followed-up for < 6 years and no endpoint events occurred, and 33 patients with MCI had no follow-up information. Our study flowchart is presented in Figure 1.

### 2.2 Participant characteristics

#### 2.2.1 Neuropsychological assessment

Neuropsychological scales were used to assess global cognition. Relevant data for the Montreal Cognitive Assessment (MoCA) were extracted from the ADNI database (see https://ida.loni.usc.edu/pages/access/studyData.jsp for details).

#### 2.2.2 18F-AV-45 PET

We utilized 18F-AV-45 PET data from a dataset of standardized uptake value ratios (SUVRs) of Aβ deposition rates in different brain regions obtained from the analysis of raw images (using the cerebellum as the reference region) at the University of California, Berkeley and Lawrence Berkeley National Laboratory (for details, see https://ida.loni.usc.edu/pages/access/studyData.jsp).

#### 2.2.3 Potential confounders

Older age is the biggest risk factor for AD (Hebert et al., 2010; 2023 Alzheimer's disease facts and figures, 2023). AD is more prevalent among women (20%) than men (10%) (Chêne et al., 2015), and there may be differences in the reasons they develop dementia, such as innate immune responses (Mangold et al., 2017; Roberts et al., 2022). Therefore, we assessed age and gender as confounders that may influence the progression from MCI to AD.

#### 2.2.4 Statistical analysis

All calculations were performed utilizing the IBM SPSS Statistics 26. Normally distributed data were expressed as mean (standard deviation), and non-normally distributed data were expressed as median (interquartile range) for continuous variables. Categorical variables were expressed as frequency (percentage, %). The categorical factors were examined using the chi-square test, while the continuous factors were evaluated by conducting the *t*-test or Wilcoxon rank-sum test in the univariate analysis.

### 2.3 WGCNA

WGCNA can be utilized to detect sets of genes with comparable expression patterns (Langfelder and Horvath, 2008). We constructed a WGCNA network for the progression from MCI to AD with all genes from the ADNI 2 dataset using the “WGCNA” R package.

#### 2.3.1 Data preprocessing

For duplicate genes, we retained the row with the highest average expression among all samples. The top 75% of median absolute deviation was screened among 19,888 genes, and a total of 14,893 genes were obtained. The “goodSamplesGenes” function was applied to detect missing values. The “hclust” function was applied to detect the outlier samples. Hierarchical clustering analysis showed that RID501, RID566, RID1406, RID4160, RID4170, RID4203, RIxD4240, RID4299, RID4426, RID4432, and RID4473 samples were the outlier samples, which were clipped and reclustered to avoid their confounding effects.

#### 2.3.2 Determine the soft threshold β

The “pickSoftThreshold” function was used to calculate different soft thresholds β for the scale-free network and the corresponding fitting exponent *R*^2^. The closer *R*^2^ is to 1, the more the fitted network conforms to the scale-free distribution, but the larger the threshold, the smaller the average connectivity of all the nodes in the network. Therefore, we determined the optimal soft threshold β to fit the optimal scale-free network based on the value of *R*^2^ and the average gene connectivity. In our study, the soft threshold β was determined to be 7 at an *R*^2^ of 0.85.

#### 2.3.3 Test whether the network constructed under the selected soft threshold β was close to the scale-free network distribution

The “scaleFreePlot” function was used to test the scale-free network.

#### 2.3.4 Obtain adjacency matrix and topological overlap matrix according to the soft threshold β

We selected β = 7 in this study, and we set the minimum number of genes contained in the module to 100. The “adjacency” function was used to obtain an adjacency matrix. The “TOMsimilarity” function was used to obtain a topological overlap matrix (TOM), and the 1-TOM was used to calculate the dissimilarity of the TOM (dissTOM). The “TOMplot” function was used to plot the correlation between the genes, and the darker the color, the stronger the interaction between the genes.

#### 2.3.5 Co-expression module identification

The dissTOM was used to construct a hierarchical clustering tree through the “hclust” function. The clustering tree was cut into different modules by the “cutreeDynamic” function. To quantify the co-expression similarity of each module, the “moduleEigengenes” function was used for calculating the module eigengene of the identified modules and the correlation of the module eigengene. We merged the modules with correlation coefficients >0.75 into one module. The “plotDendroAndColors” function was used to visualize the corresponding modules of the clustering tree.

#### 2.3.6 Correlation between modules and the prognosis of MCI

The Spearman correlation analysis was conducted; a *p*-value of < 0.05 as the correlation was statistically significant. The module with the large correlation coefficient (midnightblue module) was selected for further analysis.

#### 2.3.7 Analyze the relationship between genes and the prognosis of MCI

The midnightblue module contained many genes. For the midnightblue module, we defined module membership (MM) as the correlation of the module eigengene and the gene expression and gene significance (GS) as (the absolute value of) the correlation between the gene and the prognosis of MCI. The “plotModuleSignificance” function was used to plot the GS of the midnightblue module, and the “verboseScatterplot” function was used to plot the correlation coefficients of the MM and GS. The higher the correlation coefficient, the better. The genes that were highly correlated with the prognosis of MCI were also the core genes that were associated with the prognosis of MCI in the midnightblue module.

#### 2.3.8 Screening of key genes: in the midnightblue module

The key genes were screened according to MM > 0.8 and GS > 0.3 of the genes.

#### 2.3.9 Visualization of gene co-expression networks

The “exportNetworkToCytoscape” function was used to export the functional network information between the genes in the midnightblue module, and Cytoscape (version 3.10.0) was used to visualize the gene co-expression network.

### 2.4 Identification of differentially expressed genes

The “limma” R package was used to identify differentially expressed genes (DEGs), with the following screening criteria: a |log2fold change (FC)| of >0.263 and a *p*-value of < 0.05, where a log2FC of >0.263 and a *p*-value of < 0.05 was considered Up and a log2FC of < -0.263 and a *p*-value of < 0.05 was considered Down. The “pheatmap” and “ggplot2” R packages were used to plot the volcano and heatmap of the DEGs.

Subsequently, the obtained DEGs were intersected with the genes in the midnightblue module using WGCNA to obtain DEGs related to the prognosis of MCI.

### 2.5 Screening hub variables by machine learning

The least absolute selection and shrinkage operator (LASSO) is a data mining method that achieves an equilibrium between the model variance (the variance of regression coefficients) and the bias (the difference between the predicted value and the true value) by adjusting the parameter lambda (λ). The value of λ with the smallest error was selected as the optimal value using the 10-fold cross-validation method, and the variables included in the model corresponding to this value of λ were significant variables. The “glmnet” R packages were used for the LASSO.

The support vector machine-recursive feature elimination (SVM-RFE) constructs variable ranking coefficients based on the weight vector ω generated by an SVM during training, retains the variables with significant effects, and finally obtains the decreasing ranking of all variable attributes. In the 10-fold cross-validation method, the variables with the minimum root mean square error and the maximum accuracy were considered significant variables. The “e1072” R package was used for the SVM-RFE.

Subsequently, the significant variables obtained by the LASSO were intersected with the significant variables obtained by the SVM-RFE to obtain hub variables related to the prognosis of MCI.

### 2.6 Nomogram construction and validation

A receiver operating characteristic (ROC) curve was used to evaluate the hub variables and find the optimal cut-off value. The Kaplan–Meier analysis was conducted to compare the predicted individual risk and observe the non-progression proportion. A univariate Cox proportional-hazards regression analysis was conducted to preliminarily assess the impact of the hub variables related to the prognosis of MCI. The multivariable Cox proportional-hazards model was used to screen the independent risk factors. Hazard ratio (HR), 95% confidence interval (CI), and *p*-values were taken into account.

Based on the results of the above analysis, we found that a low MoCA score and low EBF1 expression are independent risk factors for predicting the progression of MCI to AD; meanwhile, age and gender may influence the reliability of the findings. Consequently, these independent risk factors and potential confounders (age and gender) were applied to construct the nomogram model (Li et al., 2022). The empirical samples of the ADNI 2 dataset, which were obtained by bootstrap resampling, were used as an internal validation set. External validation of nomograms is required to ensure accuracy outside the original patient data (Cote and Grassbaugh, 2024). The ADNI GO dataset was used as an external validation set. The performance of the nomogram was evaluated using the C-index and calibration curve in the training set, the internal validation set, and the external validation set. Decision curve analysis (DCA) was conducted to evaluate the clinical value of the nomogram (Zhang et al., 2018). The “autoReg,” “rms,” “bootstrap,” “pROC,” “survival,” “ggDCA,” and “rmda” R packages were used in our study.

### 2.7 Gene ontology and kyoto encyclopedia of genes genomes functional enrichment analysis

The gene ontology (GO) analysis mainly includes three parts: biological process, molecular function, and cellular component (Ashburner et al., 2000). The Kyoto Encyclopedia of Genes Genomes (KEGG) analysis provides information on gene-related signaling pathways (Kanehisa, 2002). In our study, the “clusterProfiler” R package was used for GO and KEGG functional enrichment analyses. The *p*-value was set at < 0.05, and the false discovery rate was set at < 0.25.

### 2.8 Analysis of immune cell infiltrations

We used the Cell-type Identification By Estimating Relative Subsets Of RNA Transcripts (CIBERSORT) algorithm to analyze the correlation between the EBF1 (early B cell factor 1) expression and the immune cell infiltration levels. The “CIBERSORT” R package was used in our study. The differences in immune cell abundances between the high EBF1 expression and low EBF1 expression groups were estimated using the Wilcoxon rank-sum test. The correlation between the EBF1 expression and the differential immune cells was analyzed using the Spearman correlation analysis.

## 3 Results

### 3.1 Participant characteristics

A total of 51 pMCI and 29 sMCI cases were included in the training set (ADNI 2 dataset) and 31 pMCI and 30 sMCI cases were included in the external validation set (ADNI GO dataset). The demographic characteristics, results of the MoCA, and 18F-AV-45 PET of the two sets are shown in Table 1. In the two sets, there was a statistically significant difference between the patients with pMCI and those with sMCI in the MoCA score (*p* < 0.001); therefore, the MoCA score as a variable was included in the subsequent analysis.

**Table 1:** Participant characteristics.

	**ADNI 2**			**ADNI GO**		
	**pMCI (*n* = 51)**	**sMCI (*n* = 29)**	***p*-value**	**pMCI (*n* = 31)**	**sMCI (*n* = 30)**	***p*-value**
Age	73.43 ± 7.17	70.17 ± 7.28	0.056	78.23 ± 6.99	70.30 ± 7.46	< 0.001
Women (%)	28 (54.90)	12 (41.40)	0.245	8 (25.80)	18 (60.00)	0.07
MoCA	20.27 ± 3.68	23.34 ± 2.08	< 0.001	20.84 ± 2.75	24.50 ± 2.95	< 0.001
18F-AV-45 PET (SUVR)	1.40 (0.24)	1.05 (0.26)	< 0.001	1.36 (0.23)	1.02 (0.67)	< 0.001

### 3.2 WGCNA

**Figure 2:**
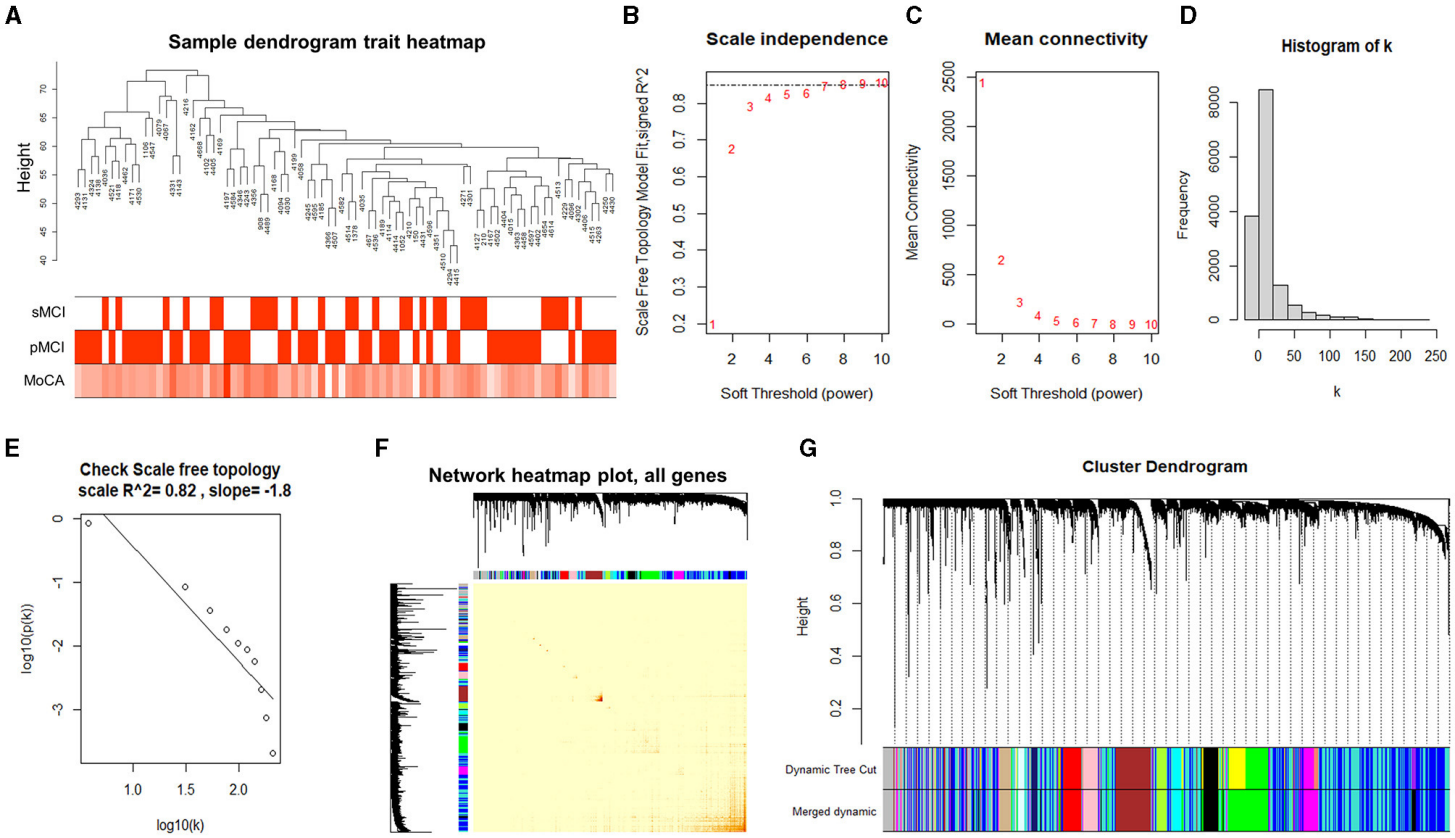
Analyzing the modules of co-expression. **(A)** Clustering dendrogram of 80 samples. **(B)** Analysis of the scale-free index for the various soft threshold powers. **(C)** The relationship between the mean connectivity and various soft threshold powers. **(D, E)** Examining the scale-free networks with the soft threshold β = 7. **(F)** The correlation heatmap between the genes in the ADNI2 dataset based on the dissTOM. **(G)** Clustering dendrogram of the genes.

After data preprocessing, we obtained the gene expression matrix of 80 samples of the ADNI 2 dataset (14,893 genes). A sample clustering tree, as shown in Figure 2A, was obtained through the clustering of the samples.

Choosing the ideal soft threshold β can enhance the robust connection and diminish the feeble connection among genes, resulting in a constructed network that closely resembles a scale-free network and is more similar to the gene regulatory network in actual biology. Therefore, we selected the soft threshold of β = 7 (using the scale-free topology criterion with *R*^2^ = 0.85) to achieve a scale-free network (Figures 2B, C).

According to the soft threshold β of 7, the histogram of the distribution of gene connectivity was plotted (Figure 2D). The scale-free network distribution was examined, which showed the number of nodes (*k*) corresponding to the gene connectivity and was negatively correlated with the probability of node occurrence (*p* (*k*)) (correlation coefficient 0.82, slope −1.8), suggesting that the network constructed by the selected soft threshold tended to converge to the scale-free network (Figure 2E).

Subsequently, we created the adjacency matrix and formed a TOM and dissTOM. The correlation heatmap between the genes in the ADNI2 dataset was plotted according to the dissTOM (Figure 2F), and hierarchical clustering was performed to merge the modules with higher similarity (Figure 2G). In the end, a total of 14 modules were discovered and the genes within each module exhibited higher similarity. The smallest module was the midnightblue module, which contained 210 genes. The largest module was the turquoise module, which contained 3,642 genes. The gray module contained all genes that could not be clustered into other modules.

**Figure 3:**
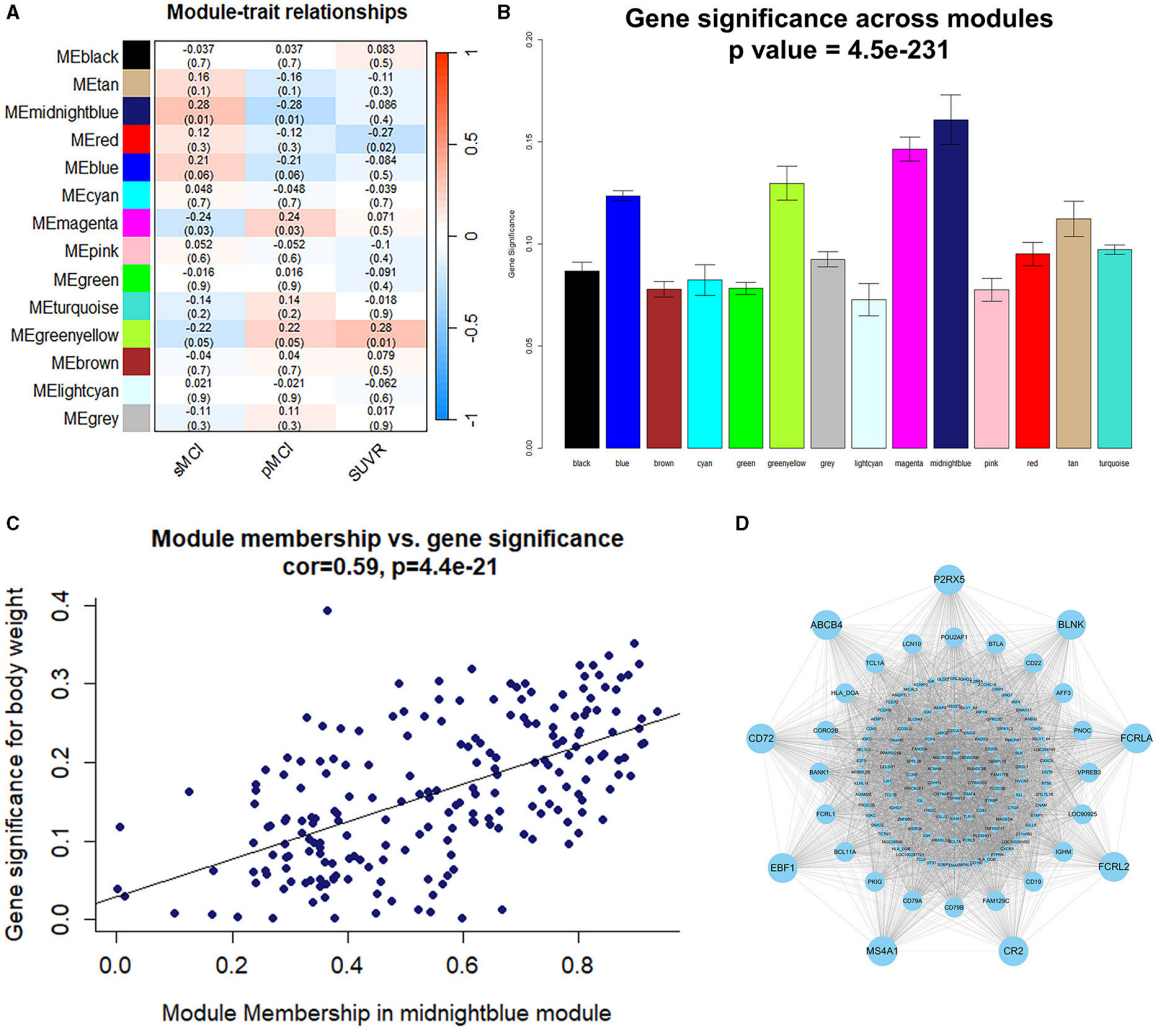
Identification of the module and genes related to the prognosis of MCI. **(A)** Heatmap illustrating the relationship between the module eigengenes and clinical status. **(B)** The correlation between the genes and the prognosis of MCI in the modules. **(C)** The correlation between the MM and GS. **(D)** Gene co-expression network and nine key genes in the midnightblue module.

The midnightblue module had a strong association with the prognosis of MCI; therefore, it was chosen as a module of clinical significance for further analysis (Figures 3A, B). Notably, a significant correlation was observed between the MM and GS of the midnightblue module (Figure 3C). By applying MM > 0.8 and GS > 0.3, we identified nine key genes (FCRLA, P2RX5, CD72, MS4A1, EBF1, BLNK, CR2, ABCB4, and FCRL2) in the midnightblue module (Figure 3D).

### 3.3 Identification of DEGs

**Figure 4:**
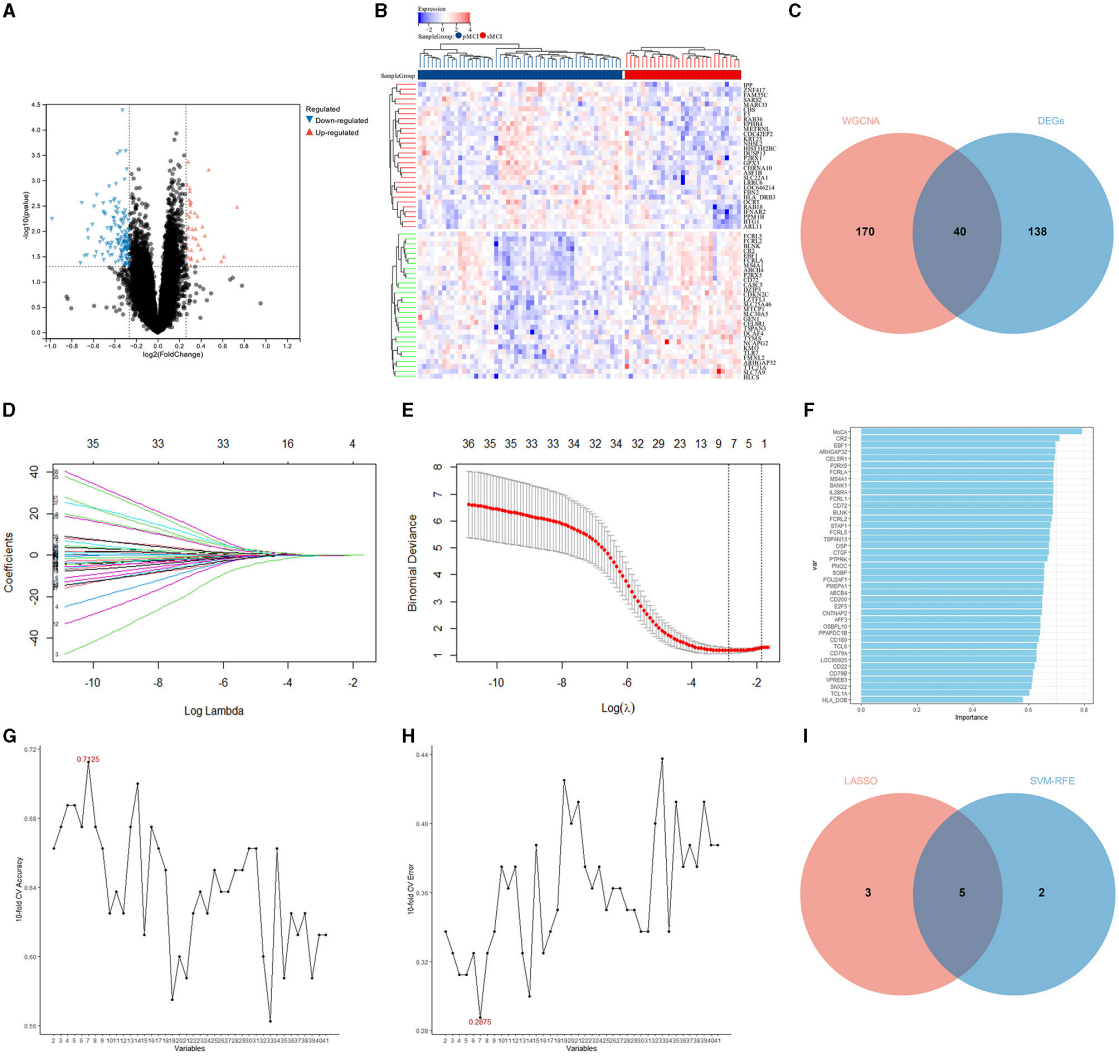
Identification of the DEGs and hub variables. **(A)** A volcano plot of the DEGs in the ADNI 2 dataset. **(B)** Heatmap of the DEGs in the ADNI 2 dataset. **(C)** Venn diagrams illustrating the DEGs related to the prognosis of MCI. **(D)** Graph showing the profiles of the LASSO coefficient. **(E)** The 10-fold cross-validation plot of the LASSO regression algorithm. **(F)** The genetic importance ranking plot by SVM-RFE. **(G)** The svm-accuracy plot of the SVM-RFE. **(H)** The svm-error plot of the SVM-RFE. **(I)** Venn diagrams of the hub variables related to the prognosis of MCI.

A total of 178 DEGs were acquired from the ADNI2 dataset, which included 114 downregulated and 37 upregulated genes. The volcano plot and heatmap of the DEGs are shown in Figures 4A, B. A total of 40 DEGs related to the prognosis of MCI were identified by taking the intersection of the DEGs and genes in the midnightblue module using WGCNA (Figure 4C).

### 3.4 Identification of hub variables by machine learning

The LASSO was performed with the MoCA score and 40 DEGs related to the prognosis of MCI, and eight significant variables were extracted, including the MoCA score, ARHGAP32, P2RX5, EBF1, FCRL2, FCRL5, CELSR1, and SOBP (Figures 4D, E).

In the optimal parameters (minimum root mean square error = 0.2875 and maximum accuracy = 0.7125), the SVM-RFE identified seven significant variables, including the MoCA score, ARHGAP32, P2RX5, EBF1, FCRLA, CELSR1, and CR2 (Figures 4F–H).

The results of the LASSO and SVM-RFE were overlapped by the Venn diagram, and finally, five hub variables were obtained, namely the MoCA score, ARHGAP32, P2RX5, EBF1, and CELSR1 (Figure 4I).

### 3.5 Independent risk factors of the prognosis of MCI

**Figure 5:**
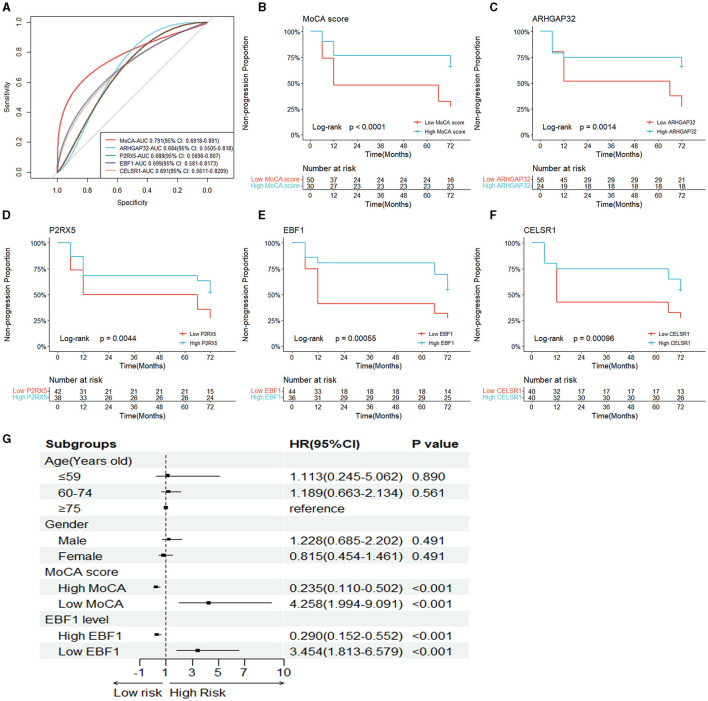
Independent risk factors of the prognosis of MCI. **(A)** The ROC curves of the five hub variables (MoCA score, ARHGAP32, P2RX5, EBF1, and CELSR1). **(B–F)** The Kaplan–Meier survival curves of the five hub variables. **(G)** Forest plot of the multivariable Cox proportional-hazards model.

To determine the ability of the MoCA score, ARHGAP32, P2RX5, EBF1, and CELSR1 to distinguish between the sMCI and pMCI and to find the optimal cut-off value, we plotted an ROC curve. The area under the ROC curve (AUC) of the MoCA score, ARHGAP32, P2RX5, EBF1, and CELSR1 were 0.791 (95% CI 0.692–0.891), 0.684 (95% CI 0.551–0.818), 0.688 (95% CI 0.570–0.807), 0.699 (95% CI 0.581–0.817), and 0.691 (95% CI 0.561–0.821) (Figure 5A). The optimal cut-off sensitivity, specificity, Youden index, positive predictive value, and negative predictive values are listed in Table 2.

**Table 2:** Classification accuracy for the prediction at the optimal risk cut-off value for the MoCA score, ARHGAP32, P2RX5, EBF1, and CELSR1.

	**AUC**	**Cut-off**	**Sen (%)**	**Spe (%)**	**Youden index**	**PPV (%)**	**NPV (%)**
MoCA	0.791	22.5	69.0	80.4	0.494	66.7	82.0
ARHGAP32	0.684	4.565	55.2	84.3	0.385	66.7	76.8
P2RX5	0.688	8.577	72.4	64.7	0.371	53.8	80.5
EBF1	0.699	5.845	69.0	68.6	0.376	55.6	79.5
CELSR1	0.691	4.631	75.9	64.7	0.406	55.0	82.5

Sen, sensitivity; Spe, specificity; PPV, positive predictive value; NPV, negative predictive value.

To assess whether the MoCA score, ARHGAP32, P2RX5, EBF1, and CELSR1 can predict AD dementia in patients with MCI, based on their optimal cut-off value, we divided the patients into the high MoCA score (≥22.5) and low MoCA score (< 22.5) groups, the high ARHGAP32 expression (≥4.565) and low ARHGAP32 expression (< 4.565) groups, the high P2RX5 expression (≥8.577) and low P2RX5 expression (< 8.577) groups, the high EBF1 expression (≥5.845) and low EBF1 expression (< 5.845) groups, and the high CELSR1 expression (≥4.631) and low CELSR1 expression (< 4.631) groups.

The Kaplan–Meier survival curves of the MoCA score, ARHGAP32, P2RX5, EBF1, and CELSR1 showed that the non-progression proportion of the patients in the low groups in the follow-up period was much lower than that of the patients in the high groups (*p* < 0.001, *p* = 0.001, *p* = 0.004, *p* < 0.001, and *p* < 0.001, Figures 5B–F).

The results of the univariate Cox proportional-hazards regression analysis showed that a low MoCA score, low ARHGAP32 expression, low P2RX5 expression, low EBF1 expression, and low CELSR1 expression increased the risk of AD dementia in patients with MCI. After the univariable analysis, the five hub variables were entered into the multivariable Cox proportional-hazards model, using stepwise backward selection, and the results demonstrated that the prognosis of MCI was significantly correlated with a low MoCA score (*p* < 0.001) and low EBF1 expression (*p* < 0.001) (Table 3). To reduce the impact of potential confounders, age and gender were also included in the final multivariable Cox proportional-hazards model (Figure 5G).

**Table 3:** Univariable Cox proportional-hazards regression analysis and multivariable Cox proportional-hazards model (stepwise backward selection) of the risk of the prognosis of MCI.

	**Univariable**		**Multivariable (backward)**	
	**HR (95%CI)**	***p*-value**	**HR (95%CI)**	***p*-value**
**MoCA score**				
High MoCA	0.253 (0.126–0.510)	< 0.001	0.226 (0.111–0.459)	< 0.001
Low MoCA	3.950 (1.963–7.949)	< 0.001	4.425 (2.177–8.994)	< 0.001
**ARHGAP32 level**				
High ARHGAP32	0.362 (0.169–0.774)	0.009		
Low ARHGAP32	2.763 (1.291–5.912)	0.009		
**P2RX5 level**				
High P2RX5	0.443 (0.248–0.790)	0.006		
Low P2RX5	2.255 (1.265–4.021)	0.006		
**EBF1 level**				
High EBF1	0.359 (0.198–0.654)	< 0.001	0.317 (0.173–0.583)	< 0.001
Low EBF1	2.783 (1.530–5.061)	< 0.001	3.154 (1.716–5.798)	< 0.001
**CELSR1 level**				
High CELSR1	0.380 (0.212–0.680)	0.001		
Low CELSR1	2.631 (1.470–4.711)	0.001		

### 3.6 Development of a nomogram prognostic model

**Figure 6:**
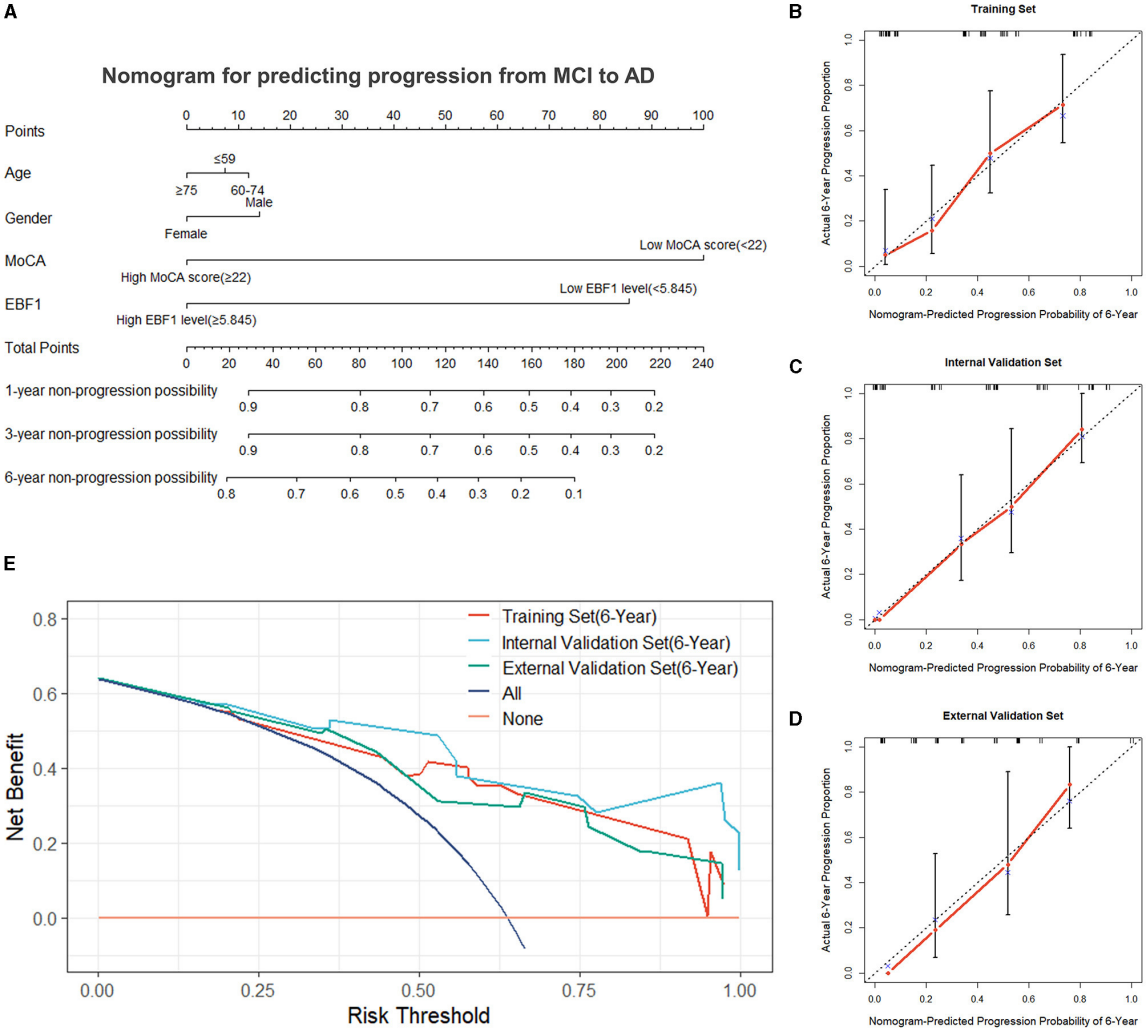
Nomogram and evaluation of the nomogram. **(A)** Nomogram was constructed for predicting the progression from MCI to AD. Calibration curves of the nomogram for the training set **(B)**, the internal validation set **(C)**, and the external validation set **(D)**. **(E)** Decision curve analysis for the training set, the internal validation set, and the external validation set.

Using the final multivariable model, a nomogram was constructed to predict the progression from MCI to AD (Figure 6A). The nomogram considered two independent risk factors (MoCA score and EBF1 expression), along with two potential confounders (age and gender). A score was assigned to each of the four variables on the point scale axis, and the scores of the variables were used to calculate a cumulative score. By projecting the total score to the total point scale, we were able to estimate the probability of patients with MCI who will progress to AD.

### 3.7 Evaluation and validation of the nomogram model

A variety of metrics, including the C-index, calibration, and DCA, were used to evaluate the performance of the nomogram. The C-index of the nomogram was 0.736 in the training set. The empirical samples of the ADNI 2 dataset, which were obtained by bootstrap resampling, were used as an internal validation set, and the C-index was 0.824 in the internal validation set. In the external validation set, the C-index was 0.751. The calibration measured how well the probabilities predicted by our nomogram model compared with the reality. Figures 6B–D present the calibration curves of the nomogram predicting a progression probability of 6 years, which demonstrated a strong agreement between the predicted probabilities by the nomogram and the actual probabilities in the training set, the internal validation set, and the external validation set. Based on the results of the DCA curves, we should avoid using our nomogram when the risk threshold is less than 13% and greater than 96% in the training set and when the risk threshold is less than 11% in the internal and external validation sets. Thus, except for a small range of risk thresholds, the net benefit of predicting progression from MCI to Alzheimer's disease in 6 years using our nomogram is greater than assuming that all patients with MCI will progress to AD or that none will progress to AD (Figure 6E). Therefore, it would be appropriate to use this nomogram in our study to predict progression from MCI to AD in 6 years. The results showed a broad spectrum of alternative threshold probabilities in the nomogram prediction model, demonstrating that our nomogram model worked well as a prediction tool.

### 3.8 EBF1 expression and function

**Figure 7:**
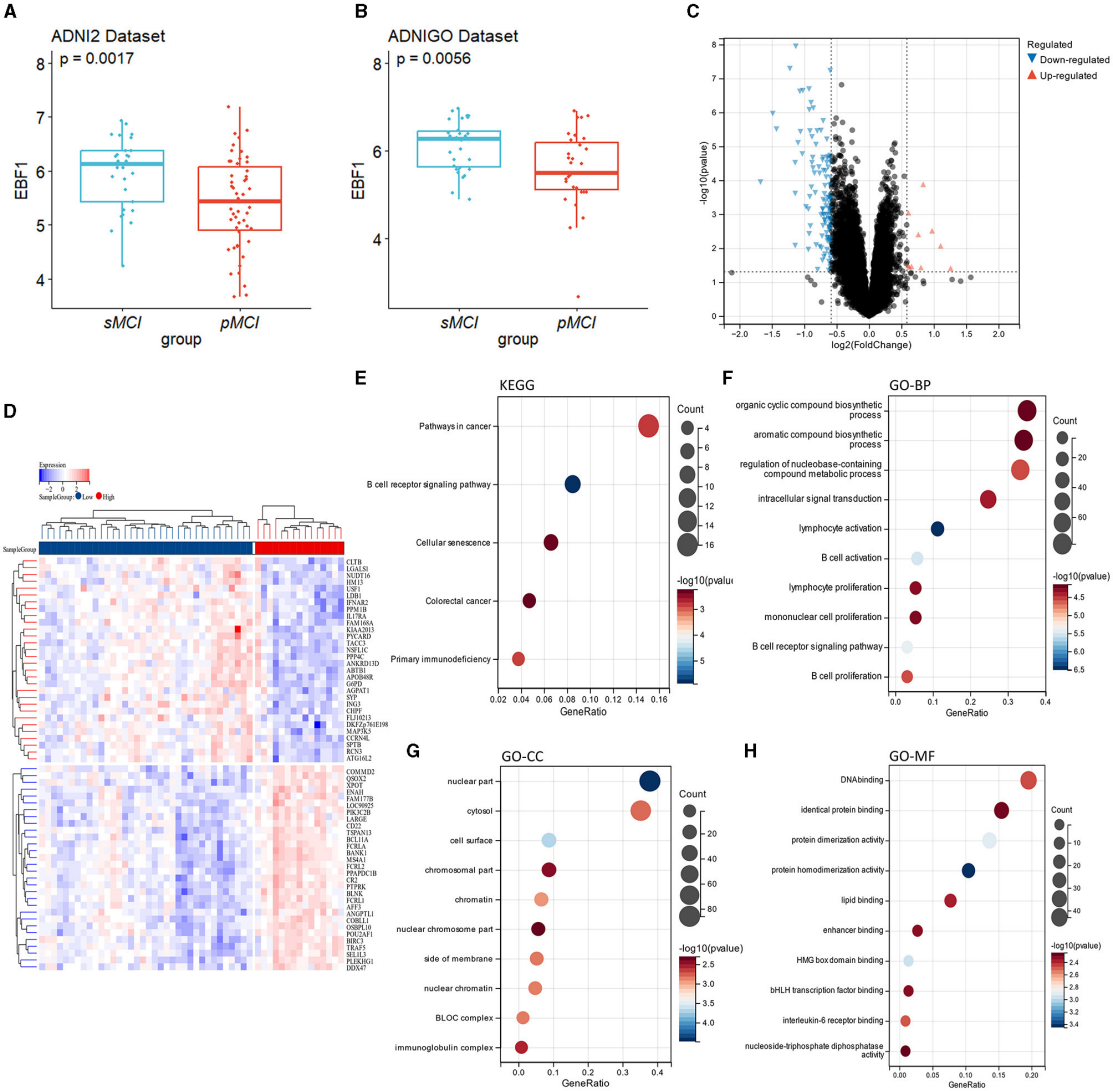
EBF1 expression and function. **(A, B)** EBF1 expression level in patients with sMCI and those with pMCI. **(C)** Volcano plot of the DEGs related to EBF1 in the ADNI 2 dataset. **(D)** Heatmap of the DEGs related to EBF1. **(E)** The results of the KEGG analysis. **(F–H)** The results of the GO analysis.

The results above showed that, in addition to the MoCA score, EBF1 is valuable in predicting progression from MCI to AD. In the ADNI 2 dataset and ADNI GO dataset, the difference in EBF1 was statistically significant between patients with pMCI and patients with sMCI (*p* = 0.002 and *p* = 0.006), and the EBF1 expression level was lower in patients with pMCI than in patients with sMCI (Figures 7A, B).

We divided the patients with pMCI of the ADNI2 dataset into the high EBF1 expression and low EBF1 expression groups. Based on the screening criteria, with a |log2fold change (FC)| of >0.263, a *p*-value of < 0.05, and a false discovery rate of < 0.05, a total of 276 DEGs related to EBF1 were acquired from the ADNI2 dataset, which included 217 downregulated and 59 upregulated genes (Figures 7C, D).

The KEGG analysis showed that the genes were concentrated in the B cell receptor signaling pathway, primary immunodeficiency, and cellular senescence signaling pathway (Figure 7E).

GO analysis includes three parts: biological process, cellular component, and molecular function. The biological process was enriched in the B cell activation, B cell receptor signaling pathway, B cell proliferation, lymphocyte activation, lymphocyte proliferation, the regulation of the nucleobase-containing compound metabolic process, and intracellular signal transduction (Figure 7F). The cellular component was enriched in the nuclear part, cell surface, biogenesis of lysosome-related organelles (BLOC) complex, chromatin, nuclear chromatin, side of the membrane, cytosol, immunoglobulin complex, chromosomal part, and nuclear chromosome part (Figure 7G). The molecular function was concentrated in the protein homodimerization activity, HMG box domain binding, protein dimerization activity, interleukin-6 receptor binding, DNA binding, lipid binding, and enhancer binding (Figure 7H).

### 3.9 Correlation between EBF1 expression and immune infiltration in MCI

**Figure 8:**
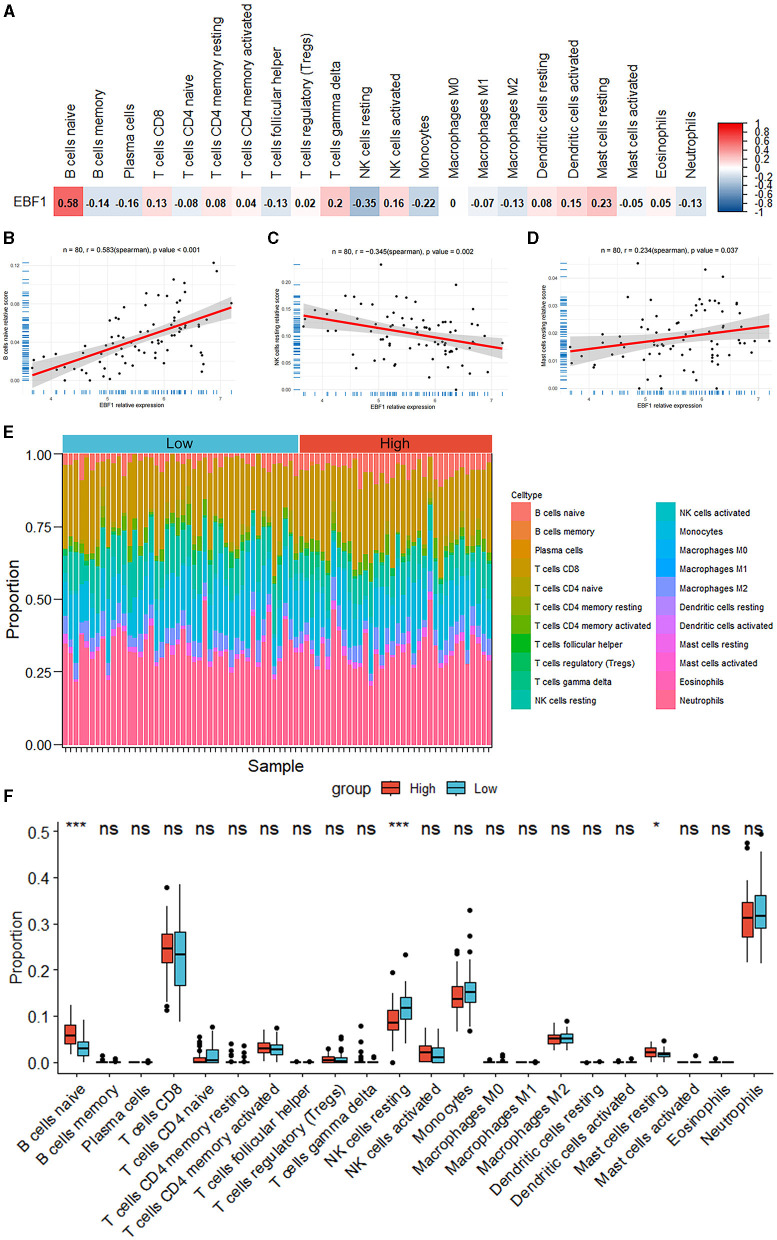
Analysis of the immune cell infiltrations. **(A–D)** The correlation between the EBF1 expression and differential immune cells. **(E)** Percentage abundance of the immune cells for each sample. **(F)** The immune cell infiltration between the high EBF1 expression and low EBF1 expression groups. ^*^p < 0.05, ^***^p < 0.001.

According to the findings of the analysis of the immune cell infiltration, the level of the expression of EBF1 was found to have a positive association with the expression patterns of B cells naïve (*r* = 0.583, *p* < 0.001) and mast cells resting *(r* = 0.234, *p* = 0.037) and a negative association with the expression patterns of NK cells resting (*r* = −0.345, *p* = 0.002) (Figure 8).

## 4 Discussion

AD is the most common type of dementia and the fifth leading cause of death in the elderly, which not only brings great pain to patients but also imposes a heavy burden on families and society. Currently, AD has a low rate of early recognition, a low rate of diagnosis, a high rate of missed diagnosis, and a high rate of misdiagnosis, and there is no treatment to reverse the disease process. The biomarkers of AD are of great significance in improving diagnostic rates and evaluating treatment, and they mainly include cerebrospinal fluid and imaging biomarkers. Currently, researchers are focused more on blood-based biomarkers that may enable earlier and faster diagnoses (Hampel et al., 2023). In addition to the plasma Aβ42/40 ratio and plasma concentrations of several pTau isoforms, new blood-based biomarkers are constantly being discovered, such as inflammation-associated glial fibrillary acidic protein and neuronal damages-associated neurofilament light chain (Cronjé et al., 2023; Jung and Damoiseaux, 2024). Meanwhile, the discovery of genetic risk factors provides a unique opportunity for a better understanding of the associated pathophysiological processes of AD (Bellenguez et al., 2022). Furthermore, Escott-Price et al. have shown that the combination of genetics and biomarkers can provide an accurate analysis in predicting the progression of AD (Stevenson-Hoare et al., 2023). However, the application of biomarkers needs to depend on the specific context-of-use, such as in low-resource and non-specialized settings, blood-based biomarkers may be more accessible, and for patients with a very likely diagnosis of AD, a cerebrospinal fluid test or a PET examination is a more appropriate choice (Parra et al., 2023). The age of the intended-use population is a critical consideration.

Machine learning algorithms could integrate multiple biomarkers for the prediction of AD. Blanco et al. (2023) found that algorithms using only fluid biomarkers have reported very good performances. As WGCNA can help researchers reveal gene co-expression patterns, discover key regulatory genes, and understand the function of gene regulatory networks, it is widely used in many areas of biological research (Gong et al., 2024; Johnson et al., 2024). For a nomogram, an externally validated and well-maintained model can be a valuable tool for predicting progression (Cote and Grassbaugh, 2024). MCI is the earliest stage of AD and is the most important target for the early diagnosis and prevention of AD. Intervention for MCI due to AD may be the most effective strategy to slow down the disease process of AD. However, the prediction of MCI to AD progression is an important clinical challenge. In our study, we applied WGCNA and machine learning strategies to hub variables related to the prognosis of MCI. Subsequently, we developed and validated a nomogram for predicting progression from MCI to AD, which provided a reliable tool for physicians for predicting the regression of patients with MCI. The nomogram consisted of two independent predictors: the MoCA score and the EBF1 gene, and it included potential confounders (age and gender). The evaluation and validation results demonstrated that the nomogram had adequate measurable power to predict the outcome of MCI.

The MoCA is an uncomplicated, independent cognitive assessment tool that demonstrates superior sensitivity. The assessment encompasses significant cognitive areas, such as immediate memory recall, delayed recall, spatial visualization skills, executive functions, verbal, abstraction, attention, numeracy, and orientation, with a total score of 30 (Nasreddine et al., 2005). Compared with the MMSE, the MoCA has a higher sensitivity for recognizing MCI and mild AD and a higher specificity for recognizing MCI (Nasreddine et al., 2005; Pinto et al., 2019; Jia et al., 2021). Our study indicated that the MoCA score has the potential to forecast progression from MCI to AD.

EBF1 is a transcription factor that regulates the differentiation of B cells, neurons, and fat cells. As a transcription factor, EBF1 was initially shown to be an essential factor for the maturation of early B cells and a key regulator of B cell gene networks (Gisler et al., 2000; Treiber et al., 2010). Studies have shown that EBF1 could regulate the development of several other cells, one of which is neurons. The EBF1 gene may be a major controller of neuronal differentiation and migration (Garcia-Dominguez et al., 2003). The findings of the current studies have qualified EBF1 as a marker gene for striatal projection neuron and early neuronal differentiation (Garel et al., 1999; Onorati et al., 2014; Mannens et al., 2024). In addition, EBF1 is a positive regulator of myelination in Schwann cells (Moruzzo et al., 2017). It was shown that etinoic acid signals could affect the migration of EBF1-expressing cells (El-Magd et al., 2014b), and the EBF1 expression could be regulated by the Shh signaling in the notochord (El-Magd et al., 2015). Although EBF1 plays an essential role in neural differentiation, its physiological function in the mature brain has not yet been identified (Lobo et al., 2006; Garel et al., 1997). The EBF1 expression was downregulated by 2-fold in the common lymphoid progenitor cells of aged mice compared to young mice (Lescale et al., 2010). EBF1 is also known to be downregulated in the *Caenorhabditis elegans* model by the co-expression of Aβ and tau in pan-neuronal cells (Wang et al., 2018).

The findings of the single-cell RNA sequencing analysis demonstrated a significant decrease in B cells in the blood of patients with AD, with the changes of the specific genes expression in B cells. The study by Xiong et al. (2021) revealed that the inactivation of B cells in the early stage significantly aggravated the AD-induced cognitive barriers with an elevated number and area of Aβ plaques in mice with AD. Recently, B cell-related processes in AD have been the subject of many studies, some of which have consistently shown that immunoglobulins produced by B cells may reduce Aβ plaques (Marsh et al., 2016) and attenuate neuroinflammation (Baulch et al., 2020). However, in a previous study, as the disease progressed in AD, B cells in mice with AD appeared to lose their anti-inflammatory activity and exhibit a pro-inflammatory phenotype, as evidenced by the upregulation of pro-inflammatory cytokine expression and the co-localization of B cells, Aβ plaques, and activated microglia (Kim et al., 2021). In summary, B cells contribute to the pathogenesis of AD and appear to play a double-edged role.

EBF1 plays a central role as a B cell-specific transcription factor in the development and maturation of B cells; thus, EBF1 is likely to be involved in AD pathogenesis. Currently, the role and mechanism of EBF1 in cognitive disorders and AD are unclear, and more experiments are needed to clarify the role of EBF1 in the future. In our study, using WGCNA and machine learning, we found that EBF1 is the hub gene related to the prognosis of MCI and is an independent risk factor for the prognosis of MCI. To clarify the role of EBF1 in MCI, our study demonstrated that EBF1 was closely associated with B cells, and an analysis of the immune cell infiltrations showed that EBF1 was most associated with B cells naïve.

By combining the EBF1 and MoCA score, we developed a nomogram, which showed an excellent ability to predict progression from MCI to AD. Our findings are meaningful for the identification of MCI due to AD and ultra-early intervention in AD. Furthermore, our predictive model is likely to be widely used because of the simplicity of the MoCA, the easy collection of blood specimens, and the low cost of these tests. However, there are some limitations to our study. First, the nomogram, and any predictive model, needs to be maintained over time, and external validation is important to improve the accuracy of the nomogram if a model truly becomes the patient counseling and decision-making tool that we want it to be (Cote and Grassbaugh, 2024). As our study is still in the preliminary exploratory stage, we need to validate the model further in multiple geographical regions, populations, and disease states for potential clinical application. Second, this was a retrospective study, so some bias was inevitable. Hence, to validate the clinical benefits, a future multicenter randomized controlled clinical study with a larger sample size may need to be carried out. Third, the prediction model was based on known risk factors, but some factors that affect MCI progress have not been studied and proven to be valid. Therefore, relevant indicators should be continuously improved in the future, which can further enhance the diagnostic accuracy of the dynamic online nomogram. Finally, our study is currently limited to *in silico* analysis. While we focus on the accuracy of our model in the clinic, we also need to clarify the role and mechanism of EBF1 in the pathogenesis of AD, which is imperative for us to explore through cell and animal experiments. This is an important direction for our future research.

## 5 Conclusion

We found abnormalities in the B-cell receptor signaling pathway by conducting bioinformatics analysis in patients with MCI who will progress to AD in the future and identified EBF1 as a potential biomarker for predicting progression from MCI to AD through machine learning algorithms and others. The analysis of the immune infiltrations showed that EBF1 was most associated with B cells naïve. Furthermore, we constructed a nomogram model (including the MoCA score, EBF1 gene, age, and gender) that was able to provide personalized risk factors for the progression from MCI to AD after evaluation and validation. We believe that our predictive models are likely to be widely used, but we still need to keep exploring and updating.

## Data Availability

The original contributions presented in the study are included in the article/Supplementary material, further inquiries can be directed to the corresponding author.
